# Percutaneous vertebroplasty combined with interstitial implantation of ^125^I seeds in banna mini-pigs

**DOI:** 10.1186/1477-7819-11-46

**Published:** 2013-02-26

**Authors:** Zuozhang Yang, Yu Zhang, Da Xu, Giulio Maccauro, Barbara Rossi, Hua Jiang, Jiaping Wang, Hongpu Sun, Lei Xu, Yanjin Chen, Xuefeng Liu

**Affiliations:** 1 Department of Orthopedics, Tumor Hospital of Yunnan Province, The Third Affiliated Hospital of Kunming Medical University, 650118, Kunming, Yunnan, P. R. China; 2 Department of Orthopedics, Peking Union Medical College Hospital, 100730, Beijing, P.R. China; 3 Department of Orthopaedics and Traumatology, Agostino Gemelli Hospital, Catholic University of Rome, Largo Francesco Vito 1, 00135, Rome, Italy; 4 Department of Radiology, The Second Affiliated Hospital of Kunming Medical University, 650118, Kunming, Yunnan, P. R. China

**Keywords:** Percutaneous vertebroplasty, Brachytherapy, ^125^I seeds, Radiation myelopathy

## Abstract

**Background:**

Although brachytherapy is one of the most effective ways to treat metastatic spinal tumor with little damage to surrounding healthy tissue, it may cause radiation myelopathy if an overdose occurs. Establishing a valuable animal model can help to find a method to overcome its complications. In the current study, we set up a banna mini-pig model to mimic percutaneous vertebroplasty with ^125^I seed implantation.

**Methods:**

Percutaneous vertebroplasty (PVP) combined with interstitial implantation of ^125^I seeds, ^125^I seeds were transplanted into the vertebral body at the T13 level of the spine in banna mini-pigs. After raising them for up to eight months, the spinal cord and vertebral body were collected for pathological analysis.

**Results:**

A potential animal model had been successfully established, no case of radiation myelopathy was found in any of the treated banna pigs, and no significant cellular impairment was noted by pathological analysis.

**Conclusions:**

It proves that PVP with ^125^I brachytherapy is an effective method to treat metastasis spinal tumor, and that the banna mini-pig can be a suitable model to investigate the mechanism of brachytherapy complications.

## Background

As the most frequent bone metastasis, spinal metastases cause severe pain and damage to vertebral bodies such as spinal osteolytic destruction and compression fractures. To avoid the trauma and complications of open surgery, a minimally invasive procedure, percutaneous vertebroplasty (PVP), has recently been developed to treat metastatic spinal tumors [1,2]. Percutaneous vertebroplasty is a newer technique in which medical-grade cement is injected through a needle into a painful, fractured vertebral body. This stabilizes the fracture, allowing most patients to discontinue or significantly decrease analgesics and resume normal activity [3-5]. In brachytherapy, a form of radiotherapy, a radiation source is permanently placed inside or next to the treatment locus. Although ^125^I brachytherapy is a better way to kill tumor cells locally and protect healthy tissues, complications can occur such as radiation damage to the tissue around the seeds, which may cause complications, especially radiation myelopathy [6,7]. How to reduce this side effect attracts abundant efforts from the relative medical researchers, but the clinically complicated situation restricts the progress of this kind of research.

It has been reported that PVP is a minimally invasive procedure with small wounds and minor complications. It is effective in the alleviation of pain in metastatic spinal tumor patients, and its clinical outcomes can be enhanced by the combination of interstitial implantation of ^125^I seeds.

An animal model has long been a good tool to help medical researchers mimic the human situation and find the best way to solve clinical problems. Aiming to solve the radiation myelopathy problem, many rodent animal models had been used, however, the rodent is small in size compared to human and their immunology system is also different from human beings, which restricts the usage of this kind of animal model. Searching for an alternative animal model is a valuable task. In this present study, we chose a banna mini-pig as our model animal; pigs have advantages over mouse models in having similar physiology to human, and comparable organ size and body mass. Pigs have a similar body size to human and their spinal cord structure also shares a similar anatomical structure with human. Aiming to mimic the actually clinical situation, digital subtraction angiography (DSA)-guided vertebroplasty was performed and ^125^I seeds were implanted into the vertebral body of banna mini-pigs. After the operation, the pigs were raised for up to eight months and, during this interval, any behavior changes were observed and written down. Pathological analysis was performed to evaluate cellular-based change. This study focused on the establishment of the PVP operation procedure with ^125^I brachytherapy and provided a good animal model for research on the treatment of metastasis spinal tumor.

## Methods

### Radiation source and reagents

Brachytherapy seeds iodine-125 (BT-125-1) were purchased from Shanghai Xinke Medicine Ltd. (Shanghai, China). Apparent radioactivity was 1.00 mCi/seed and half time is 59.4 days. Before purchase, the I-125 seeds were randomly picked up for activity testing to confirm the seed container integrity and apparent activity of the seeds.

X-ray computed tomography (CT) was from Siemens AG., Munich, Germany; DSA (digital subtraction angiography) was from Philips Medical Systems, Best, The Netherlands; the treatment planning system (TPS) was from Hejie Medical Instruments, Tianjin, China. The CRC-15R calibrator was from CAPINTEC Inc., Pittsburgh, PA, USA.

Bone cement type II was purchased from the Tianjin Material Institute, Tianjin, China. All the small instruments including sterile common needles, hammers, and seed injection needles were purchased from Guan Long Ltd., Shandong, China.

### Animals

All the animal experiments were approved by the Ethics Committee of Laboratory Animals of Tunor Hopital of Yunnan Province.

A total of 14 healthy adult female banna mini-pigs were selected for experiments. They were randomly divided into three groups, group A received the PVP with ^125^I implantation (n = 6), group B received the ^125^I implantation alone (n = 6), Group C were kept as age-matched normal control (n = 2). The animals were provided and raised by the Animal Center at Kunming Medical College. Their weights ranged from 20 to 25 kg (average 22.1 kg). The mini-pigs were raised and monitored for their food habits, excreta and activities for one week before the experiments. The protocol of the investigation was in accordance with the principles outlined in the China Practice for the Care and Use of Laboratory Animals. The pigs were raised for up to eight months (equal to four half-lives of ^125^I).

**Figure 1:**
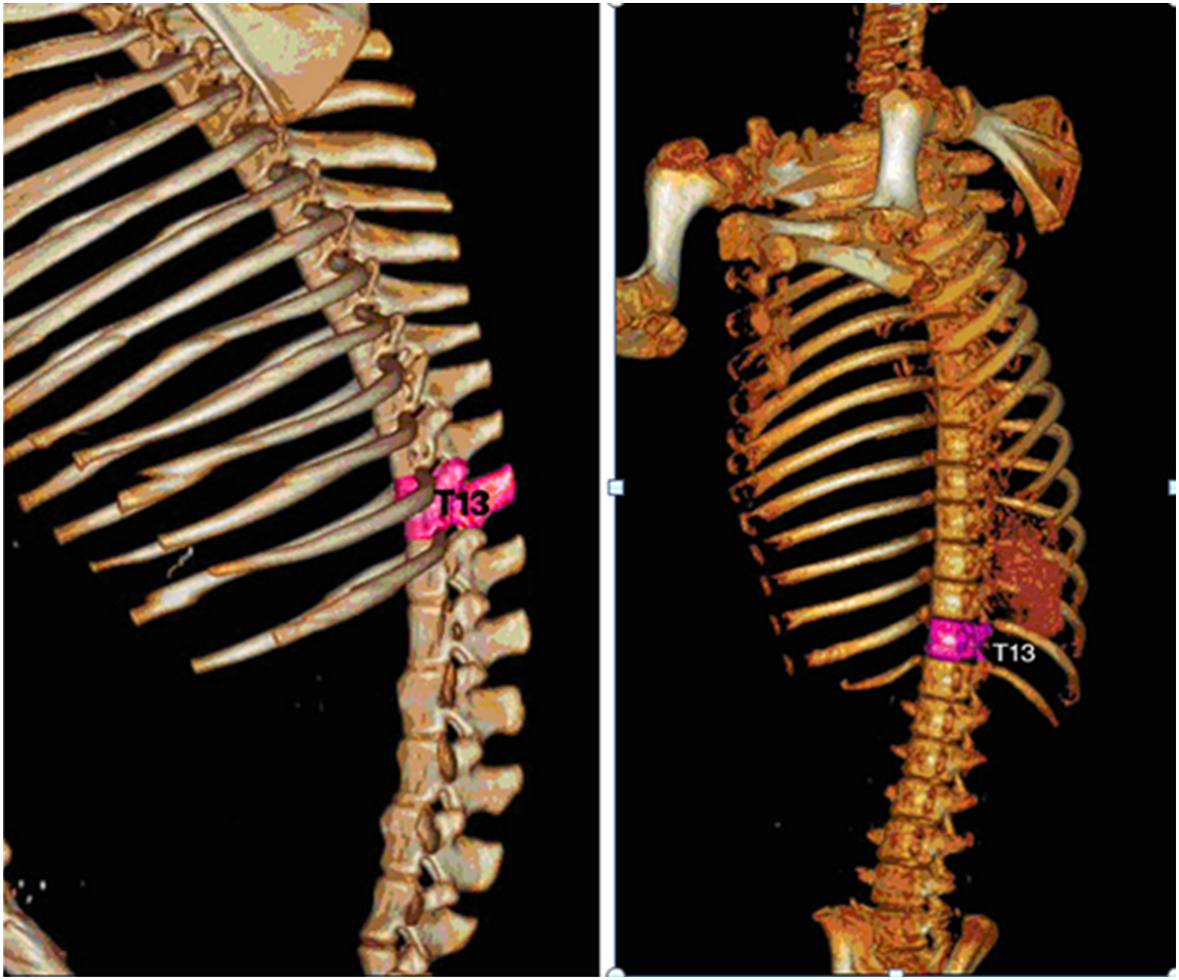
Three-dimensional schematic pictures of the T13 level of the spine in banna mini-pig.

After careful CT scans and consideration, the T13 level in the spine of the banna pig was selected as our ^125^I implanting target because it was easier to control and facilitated the whole operation. The target of the T13 level is shown in Figure 1.

### Determination of seed number for implantation

Based on the classical dosimetry calculation method cited by the Memorial Sloan-Kettering Cancer Center, the seed number was determined by the length of the three-dimensional axis, which is the distance from the seed to the target. The formula is as follows: seed number = ((length + width + thickness/3) ×5) ÷ (seed original activity), and according to the empirical experience, the actual dose density should be increased approximately 20 to 30% based on the theoretical value.

With the help of accurate CT scans, 1 cm was determined for the target length, width and thickness, the apparent activity for one seed was 1 mCi, all the data were put into formula, the predicted seed number was ((1 + 1 + 1)/3 × 5) ÷ 1 = 5, then 3 more seeds were added, so the total dosage was 8.0 mCi.

### Radiation dose calculations

This study adopted Monte Carlo-aided dosimetry to measure the radiation dose received by the mini-pig spinal cord during the whole of the brachytherapy process. Briefly, at the beginning, we calculated the initial dose (termed as D_(0)_) immediately after the ^125^I particles were implanted into the spinal cord T13 level; the formula was D_(0)_ = A_0_ × 1.27 × Λ × g_(r)_ × F_(r θ)_/r^2^. A_0_ is the particle initial radiation dose, which was tested by CRC-15R calculator on the day before implanting; Λ is constant parameter for ^125^I, in our research the value is 1.06; r is the distance between the spinal surface to the ^125^I particles, which was obtained from the magnetic resonance imaging (MRI) detecting data; g_(r)_ is radial dose functions; and F_(r θ)_ is anisotropy constant; the detailed data we calculated based on the published method [8,9]. After D_0_ had been confirmed, the radiation dose received by the spine was calculated by the formula D_(T)_ = D_(0)_*T_1/2_*1.443*(1- e^-T*0.693/T1/2^). D_(T)_ means the total received dose within time interval T, e is natural constant.

### Operation procedures

For group A, briefly, DSA-guided vertebroplasty was performed under local anesthesia, and acrylic bone cement was injected into the vertebra through a bone trocar to the center of the lesion, with simultaneous interstitial implantation of ^125^I seeds.

**Figure 2:**
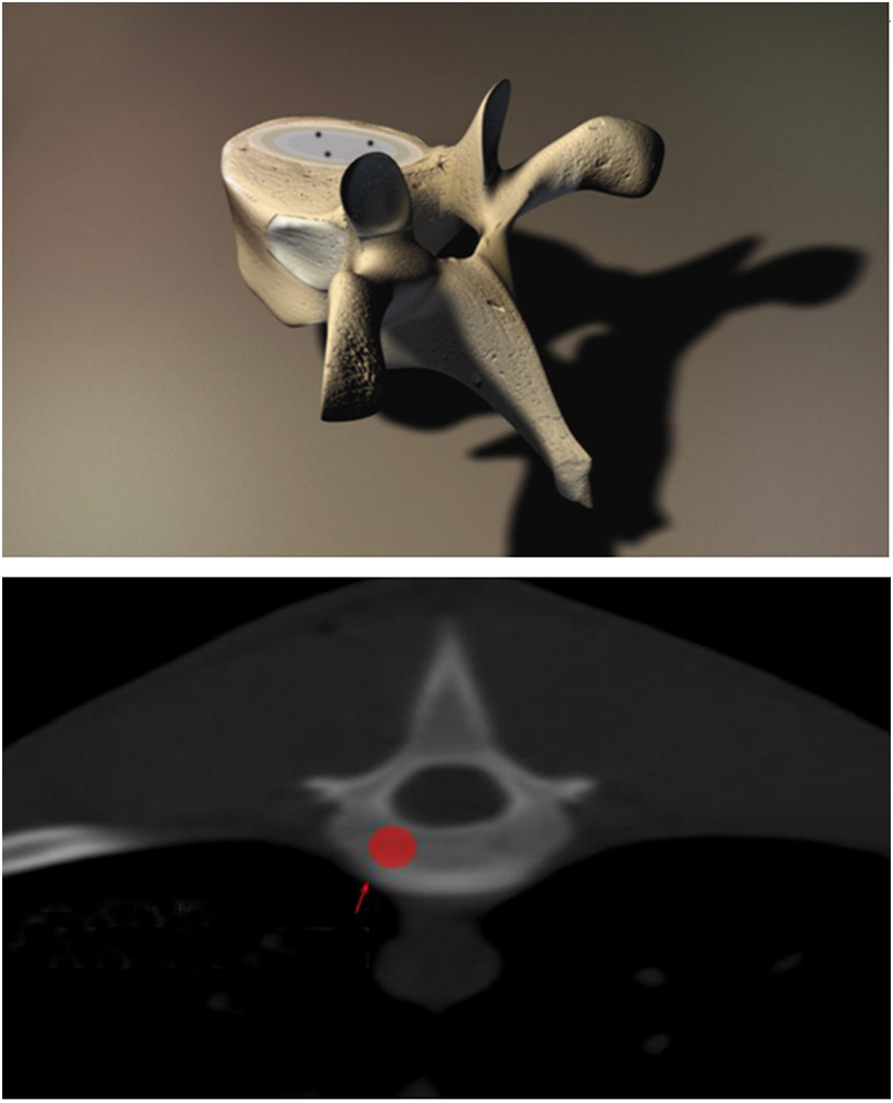
**Position in the vertebral body of PVP with the **^**125**^**I implant.**

A TPS system was implemented to manage the whole procedure. Before the operation, the T13 CT scan and the physical parameters of the ^125^I seeds were put into the TPS system to let the software create the detailed treatment plan automatically. We then followed the TPS procedure to do the operation, the mini-pigs were anesthetized with sodium pentobarbital through ear veins, and were then placed in prone positions, followed by skin preparation and sterilization. DSA was used to precisely localize the surface projection of the T13 vertebra body and vertebral pedicle. Following the template, a syringe needle, mounted with a 20 to 30 degree angle to coronal plate, went to the pedicle of the vertebral arch where it connects vertebra, and was placed into the T13 anterior spinal canal without damaging the dura mater. Meglumine diatrizoate was used to confirm the location of the needle. Then ^125^I seeds were implanted into the vertebral body. Bone cement was made up with water and photographic developer, the mixture ratio was 3:2:1 respectively, and while the cement was in the toothpaste stage, it was injected into the vertebral body, with frequent changes of direction to make the cement distribute equally and to avoid leakage. The PVP dose was approximately 0.8 to 1.3 mL, the average was 0.9 mL. After the whole operation, a CT scan was performed immediately to show the distribution of PVP and the seed locations. Figure 2 shows the detailed implant position in the vertebral body.

### Physical condition assessment of model animals

The Tarlov scale is a five-point scale to assess upper and lower limb locomotion. This scale is widely accepted by the American Spinal Injury Association (ASIA) as the International Standards for assessing spinal impairment. In this present study, the Tarlov scale was used to measure the banna mini-pigs’ spinal impairment after the operation. Hematological analyses were also performed, the peripheral blood samples were collected at five time points, they were before the seed implantation, one week, one month, two months and eight months postoperation. The white cell number, hemoglobin, blood platelet and creatinine were examined as indexes.

### Pathological analysis

After eight months, all the animals were anesthetized and executed, the T13 level was taken out to examine the filling and distribution of the bone cement and ^125^I seeds. The spinal cord and vertebral body were put into 10% formaldehyde solution for fixation. After 48 hours in 10% formaldehyde solution, the spinal cord was cut into 4mm thick slice for hematoxylin and eosin (H & E) staining, and the vertebral body remained in 1% nitric acid for further decalcification. After another 72 hours of decalcification treatment, a pin was used to test the softness of the vertebral body, if was soft, it was stained with H & E as well.

### Double staining of the spinal cord and vertebral body for electron microscopy

A series of sections from the vertebral body and spinal cord from six banna mini-pigs were processed for electron microscopic double staining to reveal cellular and subcellular alteration in morphology. For this double-labeling procedure, sections were first fixed with 3.5% glutaraldehyde solution and 1% osmic acid solution, and then dehydrated with gradient ethanol and acetone. The last step was to double stain with uranyl acetate and lead citrate, then observe under JEM-1-11 transmission electron microscopy.

### Data analysis

Standard statistical software (SPSS version11.0; SPSS, Inc, Chicago, IL, USA) was used for data analysis. Student’s *t*-test was used for variable data analysis. *P* <0.05 was considered statistically significant. Data were presented at mean ± SD.

## Results

### Confirmation of ^125^I seeds implant at the spinal T13 level by DSA and CT scanning

**Figure 3:**
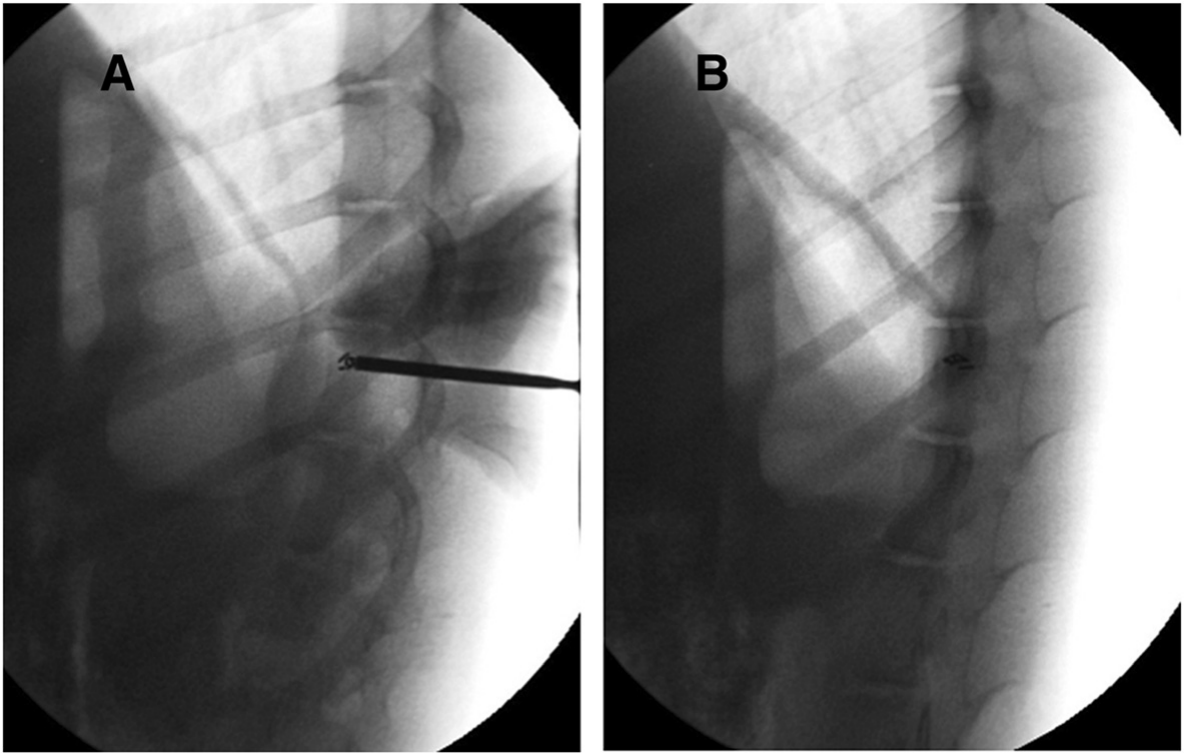
**DSA scan during the operation and postoperation. (A)** During the operation, seeds were injected into the T13 level of the spine in banna mini-pigs. (**B)** Seeds were implanted into the target region postoperation.

A DSA scan recorded the whole operation procedure, and the operation was completed successfully. See Figure 3.

**Figure 4:**
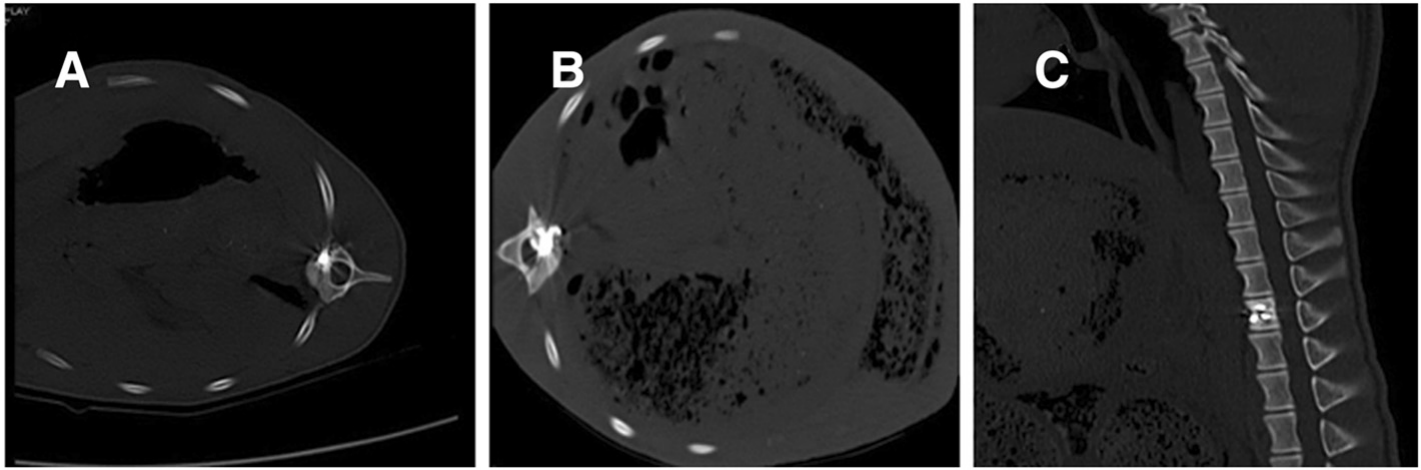
**CT scan to confirm the good distribution of bone cement and seeds in the T13 level of swine spine. (A)** and **(B)** are CT scan pictures. **(C)** is a three-dimensional schema chart of swine spine with PVP and seed implant.

After complete the operation in 15 minutes, a CT scan was performed to confirm the bone cement distribution and ^125^I seed locations. The CT scan proved the bone cement and seeds were distributed well in all the experimental animals, meeting the same requirements as human clinical standards. See Figure 4.

### ^125^I brachytherapy on day 0 postimplant dosimetry

After the operation was completed, a CT scan was performed, with the help of the TPS; the postimplant dosimetry used the dose-volume histograms (DVH) to assess implant quality. After calculation, the minimum peripheral dose (mPD) was 80.1 Gy, D90 was 90.2 Gy, the mean dose for the target region was 120.2Gy, D_90_ >mPD, suggested the seeds were successfully implanted into the correct position and the radiation dose met the requirements of brachytherapy.

### Radiation dose measurement

With the CT scan, the radiation dose distribution through the axial, sagittal and coronal planes of the vertebral body was determined in this study. Based on the formula mentioned in the Methods section, the average radiation dose to the T13 level vertebral body for each group were obtained, see Table 1.

**Table 1:** The parameters of the three dimensions of the vertebral body and the total radiation dose for the T13 level

**A (mCi)**	**r (cm)**	**g**_**(r)**_	**F**_**(r θ)**_	**D**_**(0) **_**(cGy/h)**	**D**_**(4T1/2) **_**(cGy)**	**(**$\bar{x}$**± s)**
0.39	1.03	1	1	0.50	1161.38	1014.4 ± 86.9
0.38	1.03	1	1	0.48	1035.14	
0.39	1.05	1	1	0.48	894.77	
0.41	1.03	1	1	0.52	978.49	
0.40	1.04	1	1	0.50	1004.24	
0.44	1.07	1	1	0.52	1012.38	

Note: $\bar{x}$ means the average dose that the vertebral body received in the eight months. A, particle initial radiation dose; D, total received dose; D_(0),_ initial dose, F, anisotropy constant; g_(r),_ radial dose functions; r, distance between the spinal surface to the ^125^I particles; T, time interval.

### Physical condition assessment of banna mini-pig

No paraplegia case was noted for eight months after ^125^I implantation. Tail swaying and defecating and urinating were the same as normal. The Tarlov scale was five for all the treatment animals from starting to eight months. Hematological analysis is shown in Table 2. All the data suggest banna mini-pigs had stable physical conditions within the eight months.

**Table 2:** Hematological index comparison between preimplantation and postimplantation

**Time**	**N**	**White cell (X10**^**9**^**/L)**		**Hemoglobin (g/L)**		**Platelet (X10**^**9**^**/L)**		^**creatinine **^**(μmol/L)**	
		$\bar{x}$**± SD**	***P *value**	$\bar{x}$**± SD**	***P value***	$\bar{x}$**± SD**	***P value***	$\bar{x}$**± SD**	***P value***
Preoperation	6	13.85 ± 2.09	_	140.06 ± 5.92	_	451.83 ± 14.04	_	98.82 ± 6.35	_
After 1 week	6	12.96 ± 1.31	0.432	141.80 ± 4.39	0.645	449.39 ± 16.01	0.432	99.53 ± 7.20	0.213
After 1 month	6	13.44 ± 2.12	0.523	140.59 ± 6.01	0.724	450.13 ± 16.70	0.223	98.75 ± 7.32	0.536
After 2 months	6	13.75 ± 2.02	0.642	141.40 ± 4.19	0.542	451.35 ± 14.59	0.745	97.69 ± 8.49	0.242
After 8 months	6	13.77 ± 1.72	0.589	140.98 ± 5.67	0.675	450.59 ± 15.22	0.472	97.87 ± 7.28	0.646

Note: All *P* values were compared with preoperation data.

### Pathological assessment

**Figure 5:**
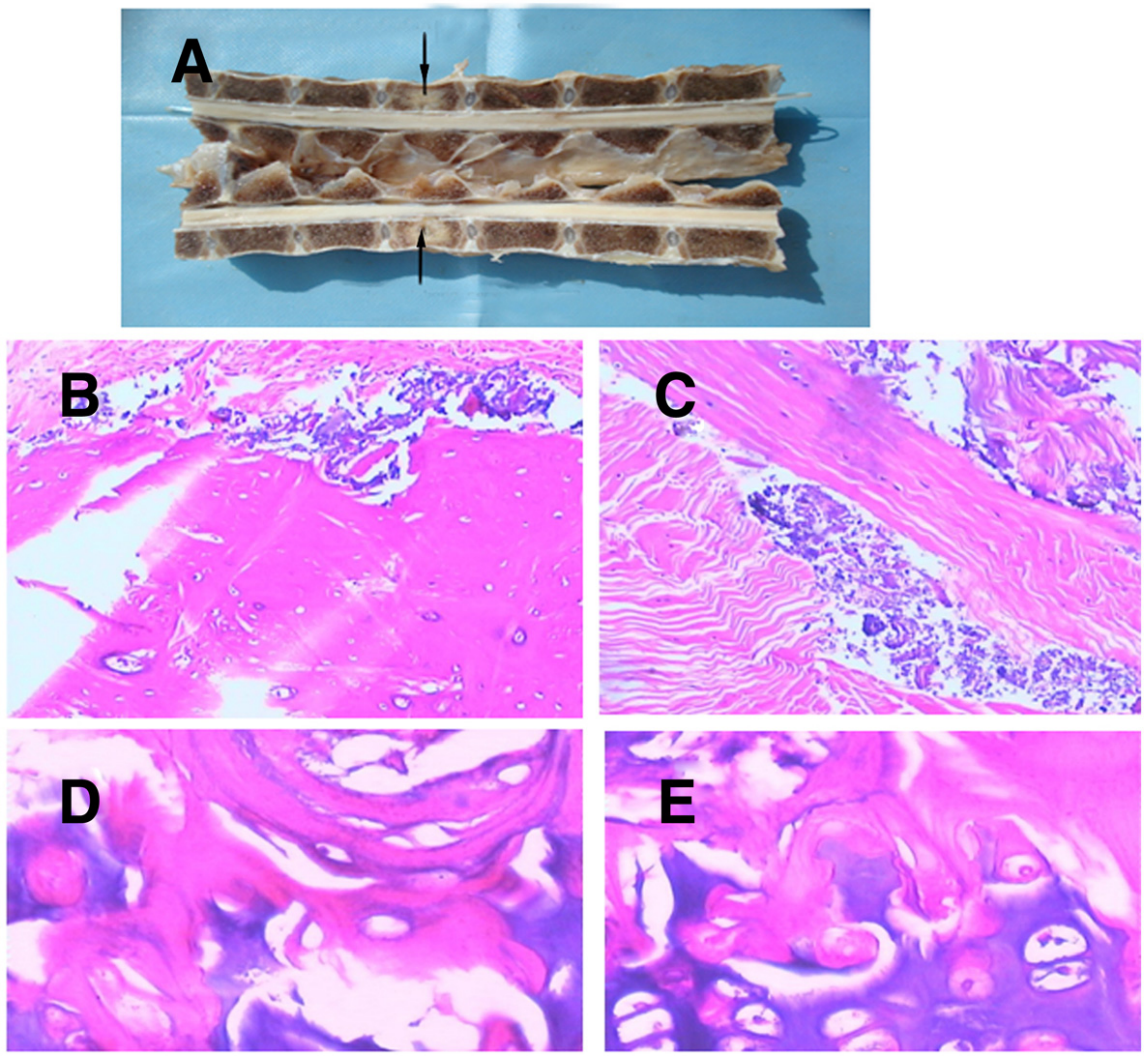
**The pathological analysis of the vertebral body. (A)** is the photograph of the half spine cut from the middle; **(B)** and **(C)** are H & E ×100 pictures, the bone cement distribution is shown; **(D)** and **(E)** were H & E stained with ×400 and ×2400 pictures, showing that the bone microstructure and bone cells keep their normal shape, no necrosis was noted.

Observation results showed the bone cement was well distributed into the vertebral body, it was almost merged together, it was so hard to separate. Under the light microscope, the bone cement was mainly distributed among bone trabeculas, stained with blue by H & E; the peripheral bone trabecula surrounding the bone cement and seeds were normal in structure; no necrosis and denature were noted for bone cells; and the morphological structure remained in normal condition as well. See Figure 5.

**Figure 6:**
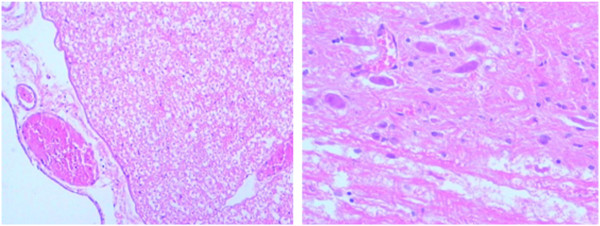
**H & E ×200 pictures for spinal cord cells. **No significant structural alteration was found.

H & E staining suggested the spine surface structure was intact; significant necrosis was not noted under the microscope; white matter was normal and grey matter had a clear structure; for neurons, shrinkage, denaturation and necrosis were not found; no swelling was observed in neuroglial cells, and microvessels in the bone were also normal. See Figure 6.

### Electron microscopic observations

**Figure 7:**
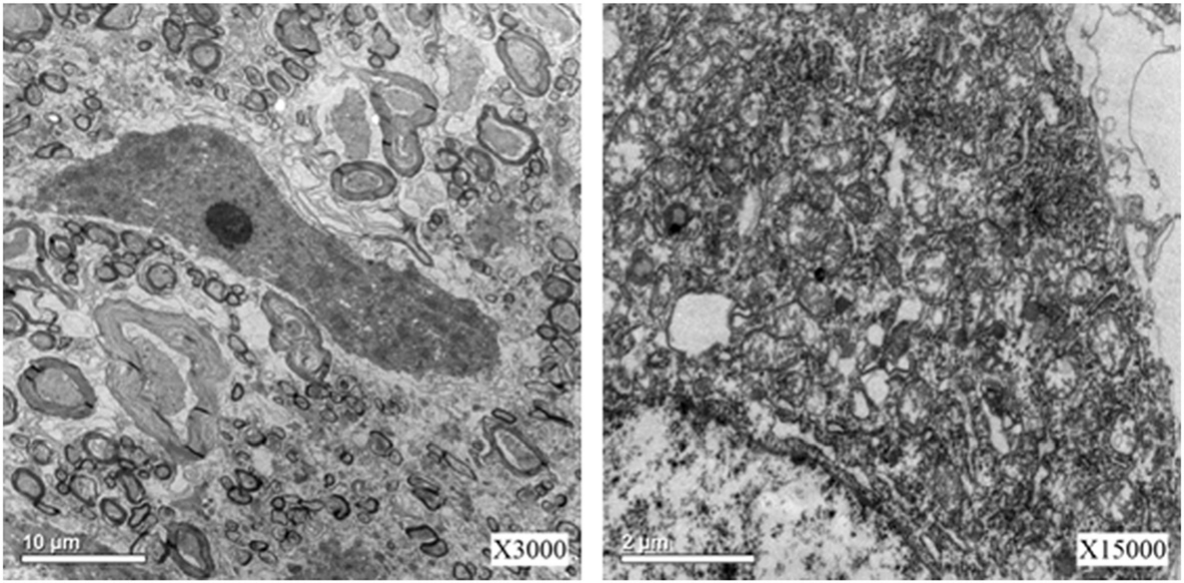
Electron microscopy observations.

Electron microscopy (EM) observation was performed for subcellular identification. Under EM, the nerve fiber structure was intact and cellular organelle was clear, no obvious impairment was found for most organelles, and only light swelling was found for mitochondria. See Figure 7.

## Discussion

Previously, we successfully established an animal model to study the pathological impairments induced by interstitial implantation of ^125^I seeds in the spinal canal of banna mini-pigs [8]. In the current study, we used this model to investigate whether percutaneous vertebroplasty combined with interstitial implantation of ^125^I seeds in banna mini-pigs could alleviate the pathological impairments. Metastases to the spine are a common problem in a large oncology population. Between 5% and 10% of all cancer patients develop spinal metastases during the course of their disease [9]. Therapeutic intervention can alleviate pain, preserve or improve neurologic function, achieve mechanical stability, optimize local tumor control, and improve quality of life. Treatment options available for metastatic spinal tumors include radiation therapy (RT), surgery, and chemotherapy [10]. The appropriate treatment for an individual patient requires a multidisciplinary review including input from a medical oncologist, internist, radiologist, radiation oncologist, neurologist, and surgeon. RT is accepted as the first-line choice for most patients with metastatic spinal tumor [11].

^125^I brachytherapy was introduced into radiation therapy in 1965 [12,13]. Brachytherapy is the placement of radioactive sources in close proximity to any tumor. It takes advantage of the simplest physical properties of radiation. High doses of radiation are present in the vicinity of a radioactive material, and a rapid drop in dose occurs with increasing distance from the source [14]. Brachytherapy has been in use worldwide since shortly after the introduction of radioactive materials. Intracavitary or surface applications are used in some human tumor types [13] (for example, cervix, skin, and bronchus), while interstitial insertions are useful in other situations (for example, head and neck, gynecologic, prostate, and sarcoma [15].

Although PVP combined with ^125^I brachytherapy has been reported to be used in clinical treatment [1,16,17], its mechanism and complications have not been investigated deeply. In this present study, we successfully established a banna mini-pig model to discuss the side effects and pathological influence of ^125^I seeds brachytherapy with PVP to neurons and other related cells. Banna mini-pig has a similar spinal structure as human, so this exploratory research will be a valuable trial for combining these two methods to treat spinal metastasis cancer in animal models.

In this study, all the geometric data related with radiation dosimetry were measured and calculated carefully, which guaranteed the radiation accuracy for this whole research; it was the most important index for this research. In this study, the average radiation dose that the T13 level received met the requirements, which simulated the actual clinical situation.

It has been reported that PVP with ^125^I brachytherapy can reduce the incidence ratio of myelopathy in clinical treatment [18]. In this study, from the animal model, we further confirm this finding. After eight months of follow-up, PVP combined with ^125^I brachytherapy did not result in any myelopathy case.

This study successfully establishes the banna mini-pig model by PVP combined with ^125^I seeds implantation and this model can help medical doctors improve operation procedure, and find a better way to alleviate patients’ pain, and it also provides a good model to study the mechanism of radiation myelopathy. We believe it will give medical doctors a good tool to improve treatment and, finally, to find a method to cure metastasis spinal tumor.

## Conclusions

It proves that PVP with ^125^I brachytherapy is an effective method to treat metastasis spinal tumor, and that the banna mini-pig can be a suitable model to investigate the mechanism of brachytherapy complications.

## Abbreviations

CT: Computed tomography;DSA: Digital subtraction angiography;DVH: Dose-volume histograms;EM: Electron microscopy;H & E: Hematoxylin and eosin;mPD: Minimum peripheral dose;MRI: Magnetic resonance imaging;PVP: Percutaneous vertebroplasty;RT: Radiation therapy;TPS: Treatment planning system

## Competing interests

All authors declare that they have no competing interests.

## Authors’ contributions

YZ and ZY was responsible for whole project design and manuscript writing; DX, MG, RB and HJ were in charge of the establishment of the animal model; JW, HS and LX were in charge of data analysis; YC and XL were in charge of data analysis and correction of the manuscript. All the authors read and approved the final manuscript.
